# Surface Finish and Residual Stresses Induced by Orthogonal Dry Machining of AA7075-T651

**DOI:** 10.3390/ma7031603

**Published:** 2014-02-28

**Authors:** Walid Jomaa, Victor Songmene, Philippe Bocher

**Affiliations:** Aluminum Research Centre (REGAL) and École de technologie supérieure (ÉTS), Département mécanique, 1100 rue Notre-Dame Ouest, Montréal, QC H3C 1K3, Canada; E-Mails: walid.jomaa@gmail.com (W.J.); Philippe.Bocher@etsmtl.ca (P.B.)

**Keywords:** machining, aluminum, surface finish, residual stress, carbide tool

## Abstract

The surface finish was extensively studied in usual machining processes (turning, milling, and drilling). For these processes, the surface finish is strongly influenced by the cutting feed and the tool nose radius. However, a basic understanding of tool/surface finish interaction and residual stress generation has been lacking. This paper aims to investigate the surface finish and residual stresses under the orthogonal cutting since it can provide this information by avoiding the effect of the tool nose radius. The orthogonal machining of AA7075-T651 alloy through a series of cutting experiments was performed under dry conditions. Surface finish was studied using height and amplitude distribution roughness parameters. SEM and EDS were used to analyze surface damage and built-up edge (BUE) formation. An analysis of the surface topography showed that the surface roughness was sensitive to changes in cutting parameters. It was found that the formation of BUE and the interaction between the tool edge and the iron-rich intermetallic particles play a determinant role in controlling the surface finish during dry orthogonal machining of the AA7075-T651 alloy. Hoop stress was predominantly compressive on the surface and tended to be tensile with increased cutting speed. The reverse occurred for the surface axial stress. The smaller the cutting feed, the greater is the effect of cutting speed on both axial and hoop stresses. By controlling the cutting speed and feed, it is possible to generate a benchmark residual stress state and good surface finish using dry machining.

## 1. Introduction

Structural aeronautic and automotive components are expected to demonstrate superior quality and enhanced functional performance. Nevertheless, the latter is strongly influenced by the surface conditions of the components. It has long been recognized that fatigue cracks generally initiate from free surfaces and that performance is therefore reliant on the surface topography/integrity produced by machining [1]. As high speed machining (HSM) is widely used in the aircraft industry due to several advantages it boasts over conventional machining, it is worth studying the integrity of dry machined surfaces. In the case of aluminum alloys, the use of high cutting speed increases metal removal rate (MRR), reduces the formation of built up edges (BUE) and burrs [2]; however, it affects the surface integrity of the machined parts [3]. It has previously [4] been shown that in the case of 7000 series aluminum alloys, fatigue resistance is primarily influenced by machining surface roughness; however, residual stresses play a second role. For machined parts made of precipitation-hardened aluminum alloys, surface roughness is considered as generating local stress concentration, and fatigue cracks were initiated on intermetallic inclusions located at the bottom of the machining grooves [5]. Thus, it is very important to understand how surface finish and residual stress state are influenced by machining of aluminum alloys.

The main difficulty in machining aluminum alloys with uncoated cemented carbide insert lies in the formation of build-up layer (BUL) on the rake surface, according to Gangopadhyay *et al*. [6]. The morphology and the mechanisms leading to the formation of BUE and BUL during machining has been the subject of several research studies [7–10]. Iwata and Ueda [8] stated that there are two types of cracks associated with BUE formation: one forms below the flank face of the tool, while the other subsequently forms ahead of the rake face of the tool. Recently, Gómez-Parra *et al*. [11] indicated that higher cutting parameter values can promote a faster formation of primary BUL in machining aerospace aluminum alloys such as UNS A92024 (Al–Cu) and UNS A97050 (Al–Zn). Moreover, the results they obtained confirmed that BUE growth is responsible for a decrease in Ra roughness [11]. Nevertheless, Iwata and Ueda [8] stated that the rearward disappearance of a BUE leaves debris containing cracks on the machined surface, and that such surface damage is undesirable because it increases the surface roughness and deteriorates the strength of the workpiece. In previous work, Gangopadhyay *et al*. [6] evaluated the performance of different cutting tools in terms of BUE/BUL formation and surface roughness during machining of AA6005 alloys. They found that the surface roughness decreases with an increase in cutting speed. The authors [6] related this decrease in surface roughness to an increase in cutting temperature, which in turn might lead to slight reduction in material adhesion. Cai *et al*. [12] studied the effect of high speed end milling on the surface integrity of 7075 aluminum alloy. Their results showed that a high cutting speed has a positive effect on the surface finish, and that residual stresses will be transformed from tensile values to compressive values when the cutting speed increases. Conversely, Ammula and Guo [13] reported that the cutting speed has a dominant effect on surface roughness, and that an increase in the cutting speed increases the arithmetic mean (Ra) during high speed milling of Al 7050-T7451 alloy. Furthermore, they found that the residual stress in the feed direction is tensile near the surface and quickly changes to compressive stresses at about 35 μm. Balkrishna and Yung [2] studied the surface integrity during the high speed face milling of 7075-T6 aluminum alloy. They reported that an increase in feed is shown to leave higher compressive residual stresses in the workpiece, while higher cutting speed and depth of cut show an opposite effect. In addition, the surface roughness improved with the cutting speed up to 1524 m/min, beyond which it showed degradation.

Based on the literature results, the machining processes that have been studied the most involved 3D setups (end milling, face milling, and turning). In these machining processes, the surface profile is strongly influenced by the cutting feed and the shape of the tool nose. Moreover, when examining the surface quality, most of the work that has been done takes into account only of the height parameters, such as the arithmetic mean (Ra), which may not fully describe the machined surface texture. Conversely, in orthogonal machining, the surface profile and roughness are influenced by the cutting conditions, the thermo-mechanical behaviour of the work materials, and the possible vibration of the machining system, rather than the geometry of the tool nose. Thus, orthogonal machining seems to provide a good indication of the inherent capability of the material to produce an enhanced/poor surface finish, regardless of the cutting tool geometry. Although several studies have been performed on the effect of surface integrity during orthogonal machining [14–17], the effect of surface finish and residual stress induced by the orthogonal and dry machining of AA7075-T651 was not addressed yet. This has therefore meant that a basic understanding of tool/surface finish interaction and residual stress generation have been lacking.

This paper presents an experimental study of the surface finish and residual stresses induced by the orthogonal dry machining of AA7075-T651 alloys. The effect of cutting conditions will be discussed. The surface topography will be analysed using two groups of surface roughness parameters: height and distribution parameters. Surface damage mechanisms were investigated in detail.

## 2. Results and Discussion

### 2.1. Surface Finish

**Figure 1.**
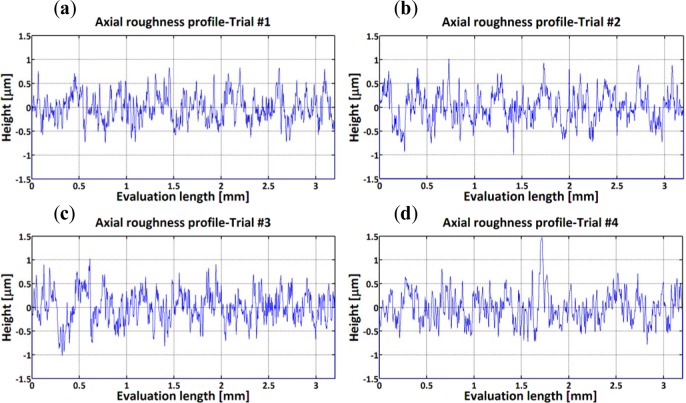
Examples of surface roughness profiles in axial direction: (**a**) Trial #1; (**b**) Trial #2; (**c**) Trial #3 and (**d**) Trial #4.

The surface roughness measurements and surface damage analysis were carried out in the representative zone. In the axial direction, 2D profiles (Figure 1) revealed grooves parallel to the tool motion.

A quantitative analysis was developed in order to quantify the effect of cutting conditions on surface topography. The height and amplitude distributions of 2D surface roughness parameters were described by the parameters given in Table 1.

Each of these roughness parameters describes one or more of the machined surface characteristics. For example, the peak-to-valley height (Rt), the mean peak to valley height (Rz), and the mean height of peak height (Rpm) parameters are sensitive to the presence of high peaks and deep scratches. The skewness (Rsk) describes the symmetry of the height distribution in relation to the mean line. On the other hand, Kurtosis (Rku) is the measure of the sharpness of the height distribution, and for a Gaussian profile, its value is equal to 3 [18].

**Figure 2.**
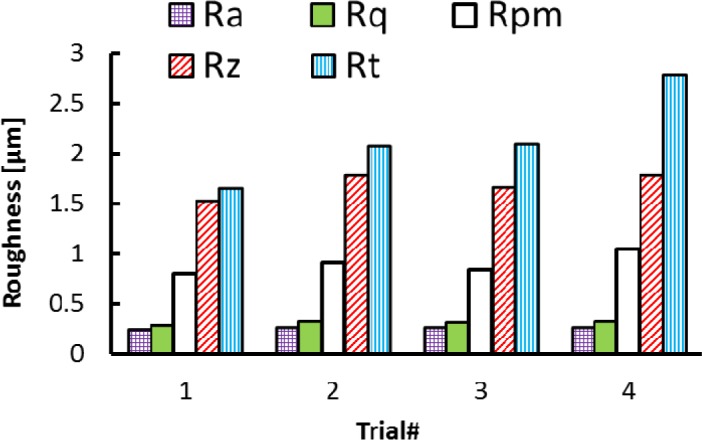
Effect of cutting conditions on height parameters in axial direction.

**Figure 3.**
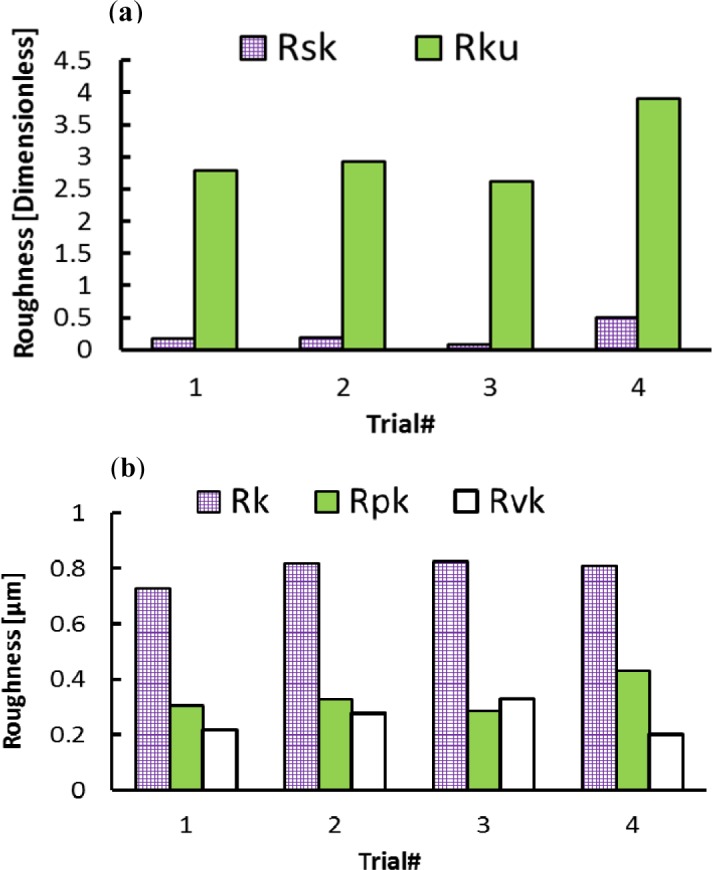
Effect of cutting conditions on amplitude distribution parameters in axial direction. (**a**) Dimensionless parameters; (**b**) dimensional parameters.

In the axial direction, the observed grooves on the machined surfaces are attributed to the ploughing effect of microbuilt-up edge BUE and the microchipping of the cutting edge. On the other hand, it can be observed that roughness parameters are influenced by the cutting feed and the cutting speed, as shown in Figures 2 and 3. The surface roughness values are the average of three measurements conducted in both the axial and hoop directions. Figure 2 show that Rt, Rz, and Rpm parameters are more sensitive and increase with the cutting feed and cutting speed, unlike the arithmetic average (Ra) and the root mean square (Rq) parameters, which change only slightly over the tested cutting conditions. Figure 3 illustrates the effect of the cutting conditions on amplitude distribution parameters. The skewness was positive and ranged between 0.078 and 0.5 (Figure 3a) indicating that the heights are symmetrically distributed about the mean line, and hence, the surface profiles were random in the axial direction [19]. The kurtosis Rku increases with the cutting feed and the recorded values were lower than 3, except for Trial #4 (Figure 3a) indicating that the distribution curve has relatively few high peaks and low valleys. Moreover, the core roughness depth Rk, which assesses the effective roughness depth after the running-in process [19,20], is slightly affected by the cutting conditions (Figure 3b). On the other hand, the reduced valley depth Rvk and the reduced peak height Rpk increases with the cutting feed when machining at lower cutting speeds (Trials #1 and #2). However, when machining at higher cutting speeds (Trials #3 and #4), the Rpk decreases and the Rvk increases with the cutting feed (Figure 3b).

**Figure 4.**
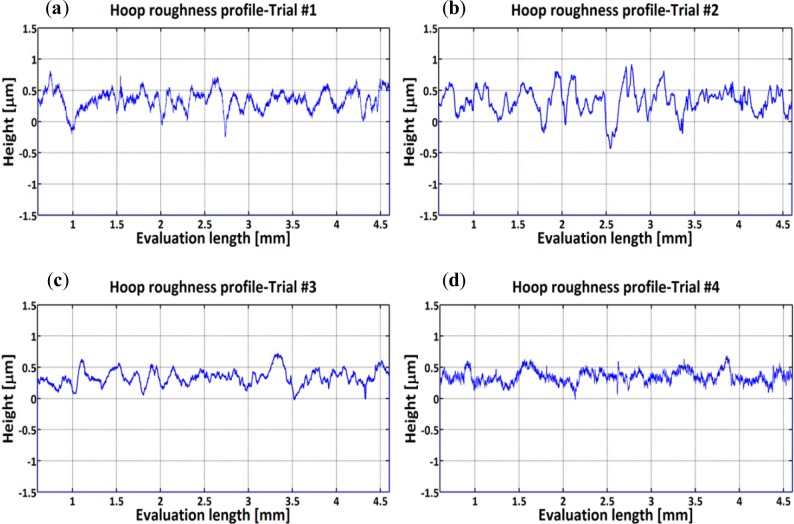
Surface profiles in hoop direction: (**a**) Trial #1; (**b**) Trial #2; (**c**) Trial #3 and (**d**) Trial #4.

**Figure 5.**
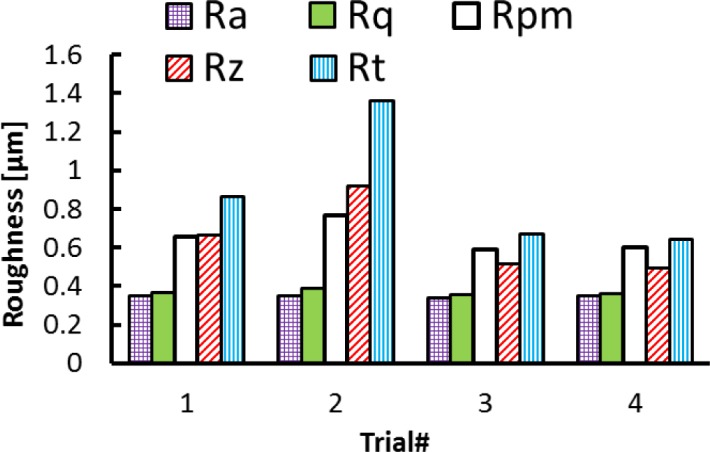
Effect of cutting conditions on the height roughness parameters in hoop direction.

**Figure 6.**
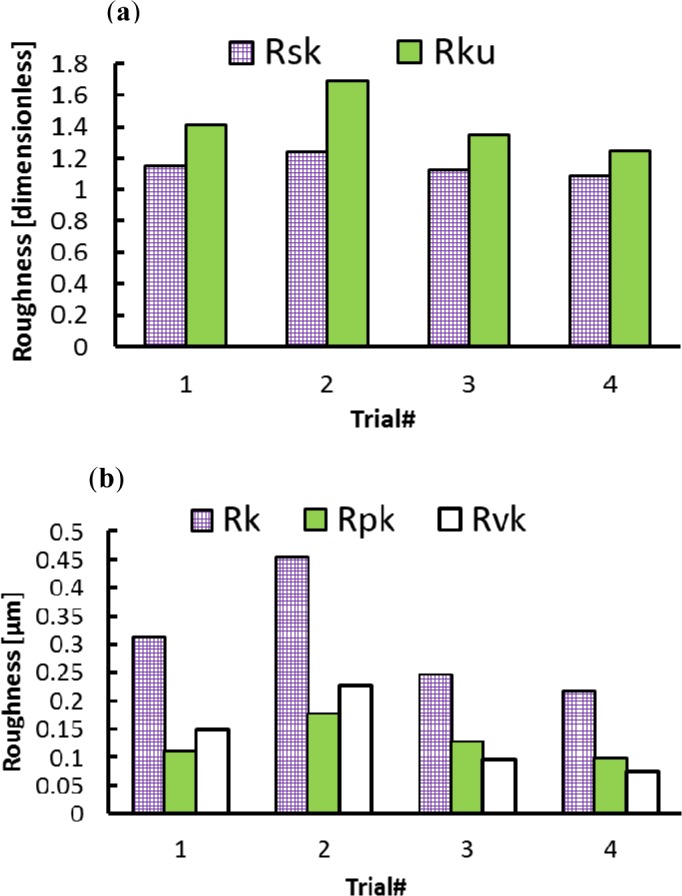
Effect of cutting conditions on amplitude distribution parameters in axial direction: (**a**) dimensionless parameters; (**b**) dimensional parameters.

Distinguishing features can be identified in the hoop direction as cutting conditions change (Figure 4). Sharp peaks and deep valleys are produced at lower cutting speeds (Figure 4a,b) and blunt irregular peaks are seen when machining at higher cutting speeds (Figure 4c,d). Figures 5 and 6 present the effect of cutting conditions (cutting speed and cutting feed) on surface roughness parameters measured in the hoop direction. The highest surface roughness values were obtained in Trial #2 where a cutting feed of 0.25 mm/rev and cutting speed of 300 m/min were used. Furthermore, for both groups of roughness parameters analysed here, the values increase with the cutting feed and decrease with the cutting speed. This result is in agreement with those obtained when machining using standard cutting operations such as turning and milling [21,22].

In the hoop direction, the skewness ranged between 1.089 and 1.236, which once again indicates the non-random aspect of the surface profile. In addition, the Rku values were lower than 3 for all tested conditions indicating that only relatively few high peaks and low valleys were found [23].

**Figure 7.**
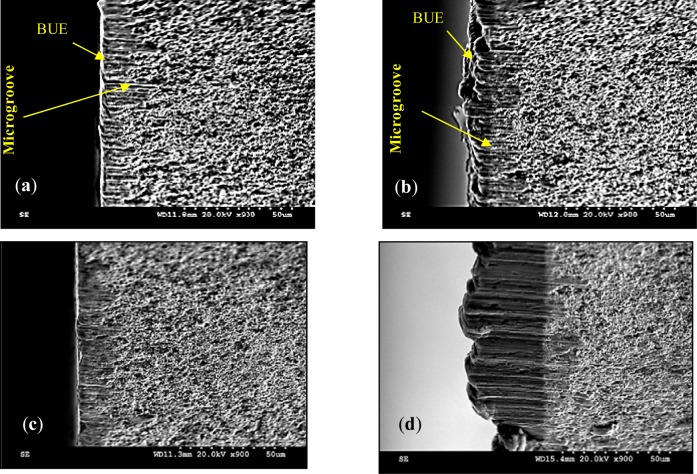
Flank face of the cutting tool after machining at (**a**) Trail #1; (**b**) Trial #2; (**c**) Trial #3; and (**d**) Trial #4.

**Figure 8.**
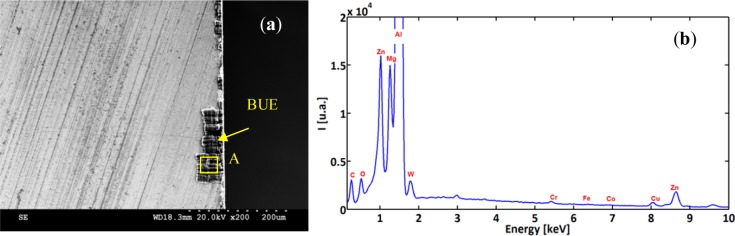
(**a**) Rake face of the cutting insert for Trial #2 and (**b**) EDS spectra acquired on BUE-Detail A.

**Figure 9.**
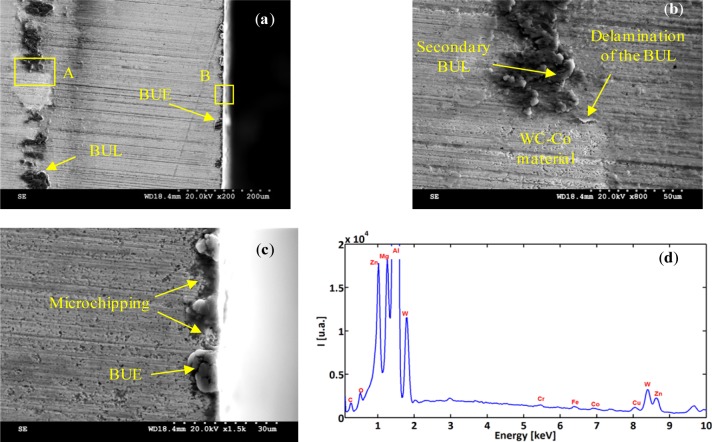
(**a**) Rake face of the cutting insert for Trial #4; (**b**) BUL-Detail A; (**c**) micro-chipping of the tool edge Detail B and (**d**) EDS spectra acquired on secondary BUL.

To investigate the tool/work material interaction during machining and its effect on the surface quality, SEM analyses were performed on both the cutting tool and the machined surface. Figures 7 and 8 illustrate the formation of the BUE and BUL on the cutting tool. The formation of BUE was intensified by increasing the cutting feed (Figure 7). The presence of microgrooves on the BUL that formed on the flank face could be related to the hard intermetallic phase present in the T6 condition of the alloy [24]. However, an increase of the cutting speed reduces this phenomenon and promotes the formation of the BUL on the rake face, as can be seen in Figure 9. Figure 9a shows a thin layer, which is considered as the primary BUL, on the rake face. Moreover, depending on the cutting conditions, the tool-chip contact area can be divided into three regions: (a) first sticking zone, close to the edge; (b) a sliding zone and (c) second sticking zone at the rear end of the contact (Figure 9a) [25]. The sticking area starts to develop and enlarge as the cutting speed increases, and results in a secondary BUL (Figure 9b). In fact, when dry machining aluminum alloys, the temperatures in the chip-tool interface is high enough to soften the aluminum matrix and it can thus provoke the adhesion of quasi-pure aluminum to the tool rake face [10,11]. Increasing the feed and speed involves increasing in the intensity of the adhesion effects [26]. These results agree with those obtained by Gangopadhyay *et al*. [6] in the case of dry machining of AA6005 in a cutting speed range of 200–1000 m/min. The high degree of chemical affinity of aluminum alloy towards cemented carbide (composite of WC and Co) is thought to be the primary reason for this phenomenon [27].

**Figure 10.**
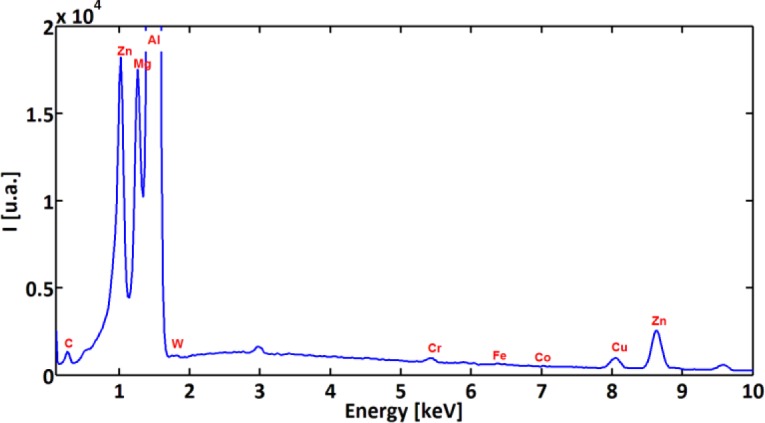
EDS spectra acquired on a machined surface.

In order to characterize and distinguish the morphology differences between BUL and BUE, EDS analysis of both zones was carried out. Figures 8b and 9d display the EDS spectra acquired on zones corresponding to BUE (A) and BUL (B), respectively. As a reference, an EDS spectrum acquired on a machined AA7075-T651 alloy is also reported (Figure 10). The EDS spectra are quite different in particular for the intensities of the Mg, Fe, and W peaks. They are lower in the BUE than those in the BUL. This could point out the dissimilar nature of the BUE and BUL regions [10]. In fact, the high temperatures reached in the initial stages of the cutting process cause the incipient melting of the Al matrix in the alloy, which flows on the rake face of the tool [9]. Under these conditions, the metallic chips would drag off the hard intermetallic particles and a part of these particles will attach to the second sticking zone, leading to high amounts of Fe and Mg elements on the secondary BUL. In addition, when the cutting speed increases, the BUE increases to a critical thickness, after which it is plastically extended over the BUL [10] and/or broken due to the action of mechanical forces. This cyclic phenomenon induces the microchipping of the tool edge, and, therefore, W-riche particles will be dragged off by the chip and adhere to the secondary BUL leading to a higher percentage of W as compared to the BUE. The broken BUE is not only evacuated by the chip but, depending on the cutting conditions, it can be squeezed under the cutting edge, causing damage to the new machined surface. Moreover, the disappearance of the BUE results in debris adhered to the machined surface, which degrades its roughness [8]. This can explain the enhancement of the surface finish as the cutting speed increases [25], as stated above.

**Figure 11.**
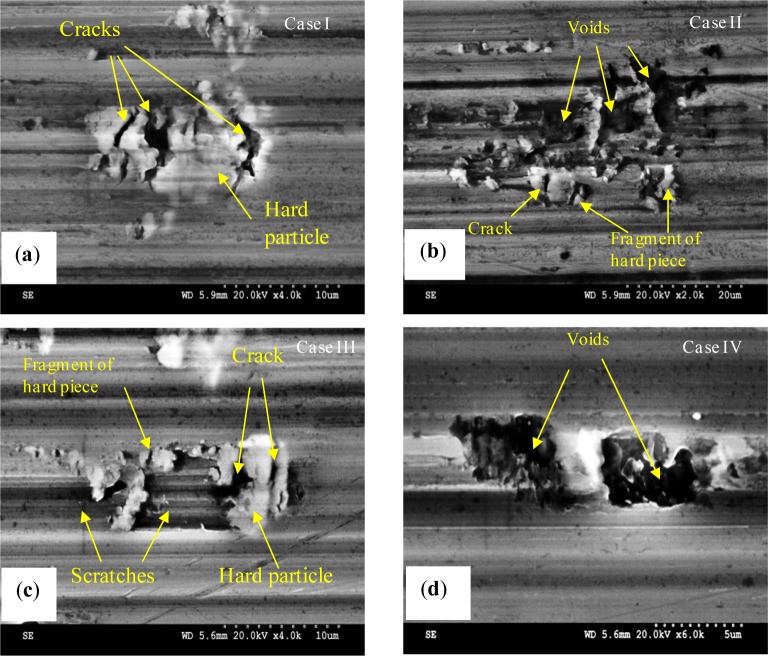
SEM micrographs of the machined surfaces showing (**a**) hard particle cracking; (**b**) smearing; (**c**) cracking and dragging of hard particles and (**d**) voids. (**a**) and (**b**) for Trial #2 and (**c**) and (**d**) for Trial #4.

**Figure 12.**
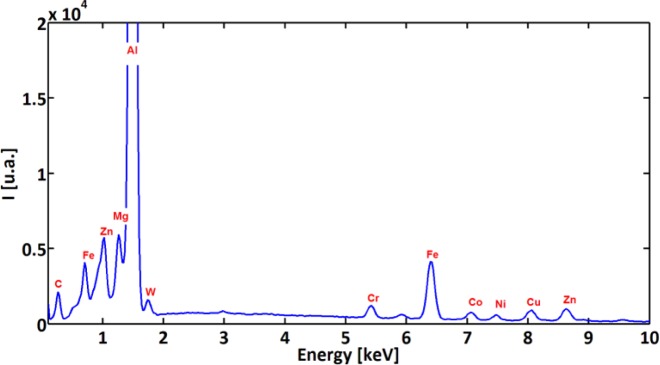
EDS analysis of the hard particle.

A close examination of the machined surface using SEM reveals a different type of surface damage. Figure 11 shows that surface damage was produced by the interaction between the cutting tool and hard particles present within the work material matrix. The different forms of damage documented here were cracks of the hard particles (Figure 11a), smearing (Figure 11b), dragging of hard particle (Figure 11c), and voids (Figure 11d). These particles are composed mainly of iron-rich intermetallic phase as shown by the EDS spectra (Figure 12) [24].

**Figure 13.**
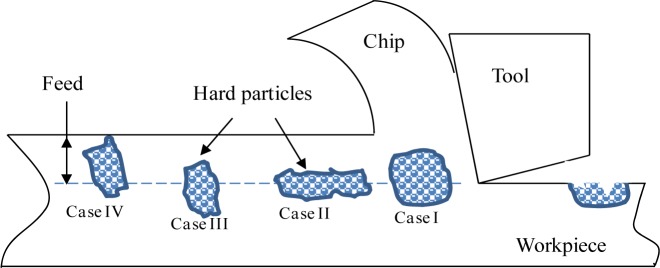
Illustration of mechanisms of surface damage induced by tool/hard particle interaction.

Depending on the cutting conditions and the relative position of these particles with regard to the tool edge, four cases of surface defects can be distinguished (Figure 13). If the hard particle is large enough and the volume embedded in the matrix is comparable to that located above the tool edge path (Figure 13, Case I), then the particle will crack and some of theme remain attached to the surface (Figure 11a). When the tool comes into contact with a large but thin hard particle (Figure 13, Case II), the latter breaks up into small parts, resulting in a smearing, as shown in Figure 11b. When the hard particle is long enough and the volume embedded in the matrix is higher than that located above the tool edge path (Figure 13, Case III), then the latter will crack and some of the cracked particles will be dragged, resulting in a grooved surface (Figure 11c). Finally, case IV is similar to case III, but the volume embedded in the matrix is lower than that located above the tool edge path (Figure 13-Case IV). In this case, the hard particle will break, and the fragments will be removed from the surface, leading to surface voids, as shown in Figure 11d.

**Figure 14.**
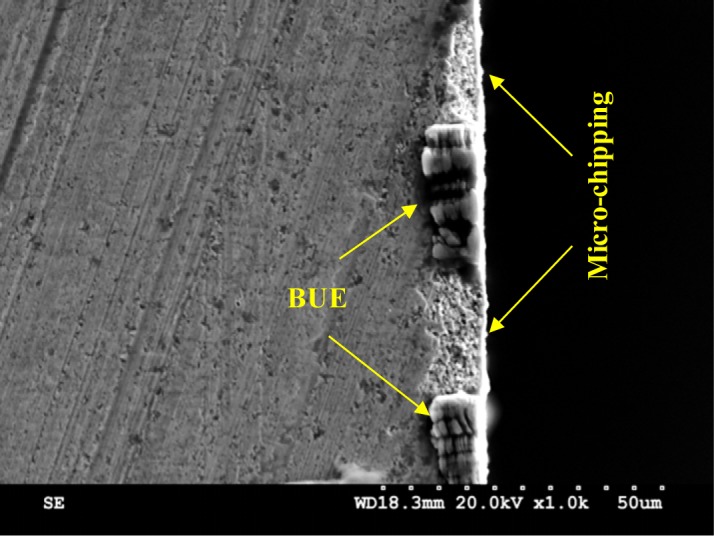
SEM image of the cutting insert for *f* = 0.25 mm/rev and *V* = 300 m/min.

It should be noted here that, based on the SEM images, no quantitative conclusion could be made about the effect of cutting parameters (feed and speed) on the generation of these damages. However, we can argue that hard particles together with the BUE formation are the primary sources of the micro-chipping of the cutting tool edge (Figure 14).

In the present study, the analysis of the surface topography showed that the surface roughness values were not zero as expected, and were sensitive to changes in cutting parameters (cutting feed and speed). Surface roughness was found to be influenced by the formation of BUE and the interaction between the tool edge and the iron-rich intermetallic particles. Moreover, the effect of cutting speed and cutting feed on height roughness parameters (such as Rt and Rz) depend on the measurement direction (Axial or hoop). Both cutting speed and feed increases the height roughness measured in the axial direction. This suggests that the micro-chipping of the cutting tool edge is intensified when the cutting feed and cutting speed were increases. On the other hand, the appearance of surface damages, such as cracks, voids and smearing is governed by a complex phenomenon including the degree of material softening, cutting forces and cutting temperature. These defects could be sites of failure initiation [28] and that surface roughness measurements are not sufficient to determine the surface conditions [29]. In addition, adjusting cutting parameters according to these defects is very hard, and even then, a complete elimination is not possible [30].

### 2.2. Residual Stress

**Figure 15.**
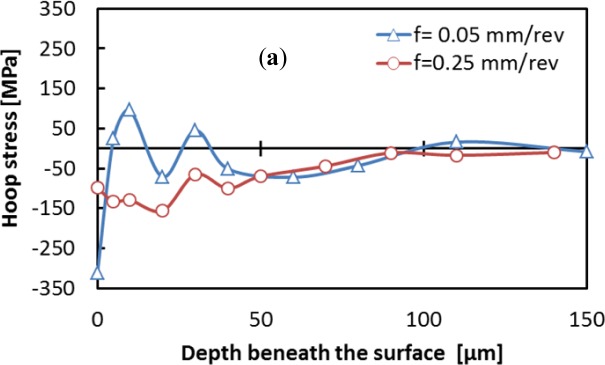

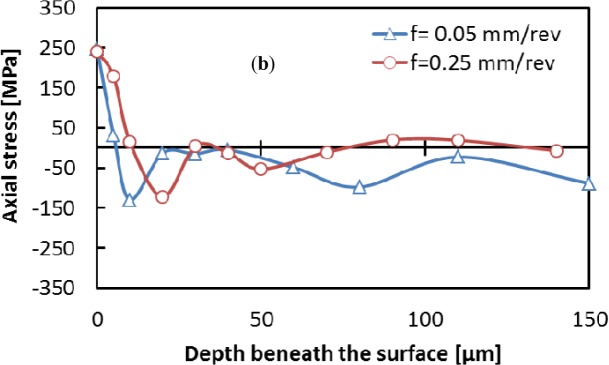
Effect of cutting feed on (**a**) hoop and (**b**) axial residual stress distribution for cutting speed of 300 m/min.

**Figure 16.**
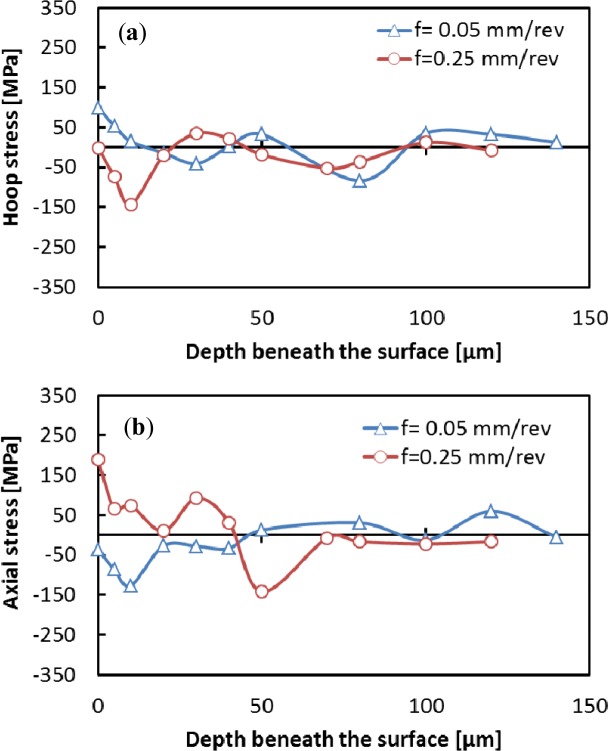
Effect of cutting speed on hoop (**a**) and axial **(b**) residual stress distribution for cutting speed of 1000 m/min.

**Figure 17.**
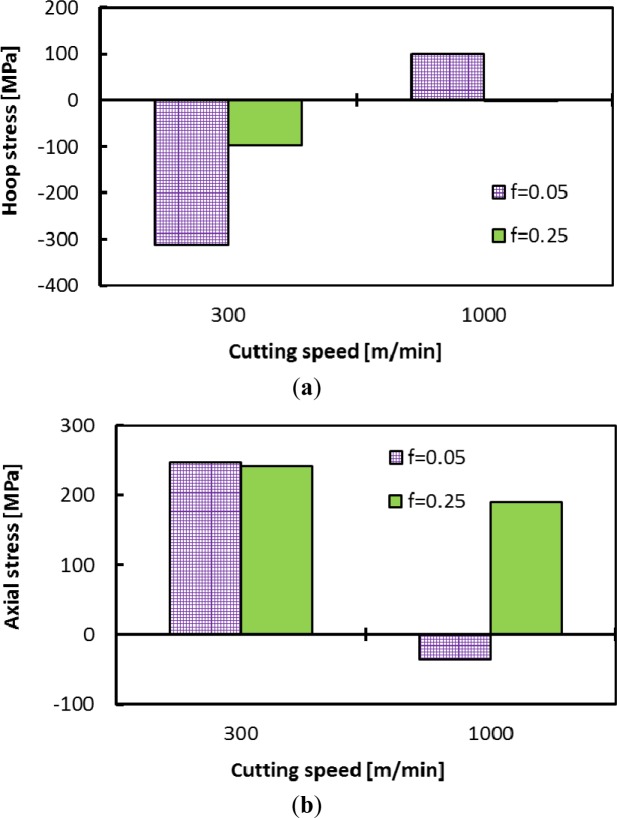
Effect of cutting speed on hoop (**a**) and axial (**b**) surface residual stresses.

The machining induced residual stresses are very important parameters that should be considered in the design of mechanical parts. This section focuses on the effect of cutting conditions on the residual stress distribution. The residual stress distribution in both the hoop and axial directions were measured and are shown in Figures 15 and 16. The residual stress values increases or decreases from an extreme level at the surface, and fluctuates along the measured depth. These results are in agreement with previous work [12] and could be related to the coarse grain microstructure of the aluminum alloy. Surface hoop stress was predominantly compressive for low cutting speed (Figure 17a). However, axial stresses tends to be tensile when cutting speed increases (Figure 17b). In addition, the smaller the cutting feed, the greater the effect of cutting speed on both axial and hoop stresses.

**Figure 18.**
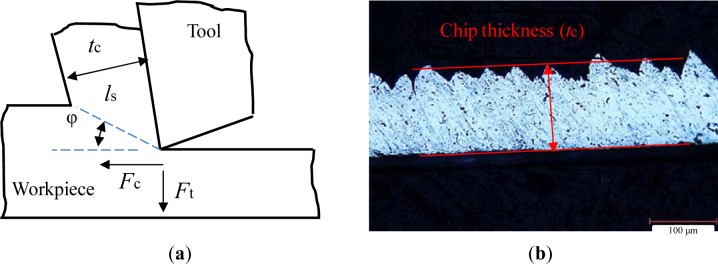
(**a**) Illustration of the orthogonal machining; (**b**) example of an optical image of the chip for Trial #1.

The residual stresses could be interpreted using geometrical parameters of the cutting zone as proposed by Liu and Barash [31]. The shear plane length is the fundamental parameter that governs the mechanical state of the surface (for both residual stresses and plastic deformation) since it is related to the frictional and shearing processes of the chip removal. According to the cutting mechanic, the shear plane length (*l*_s_) is inversely proportional to the shear angle (φ) (Figure 18a). The shear and friction angles (φ and β, respectively) were calculated, based on the measured cutting forces (*F*_t_ and *F*_c_), chip/tool contact length (*l*_c_), and chip thickness (*t*_c_) (Figure 18b), and using the well-known Merchant’s cutting theory. The measured and calculated results were presented in Table 2.

**Figure 19.**
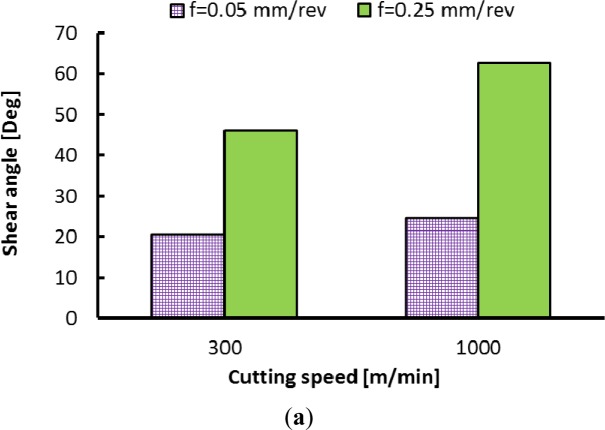

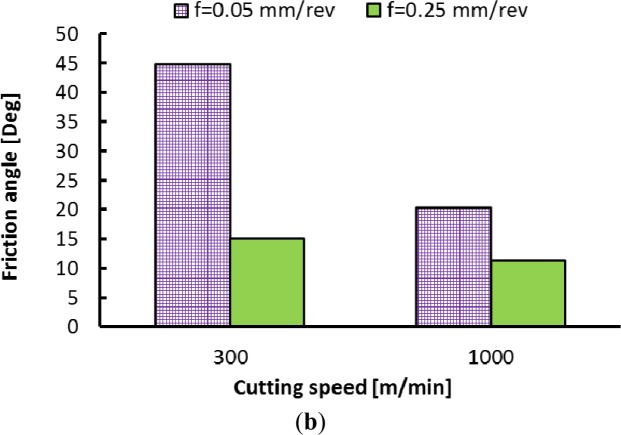
Effect of cutting speed on (**a**) shearing and (**b**) friction angle.

**Figure 20.**
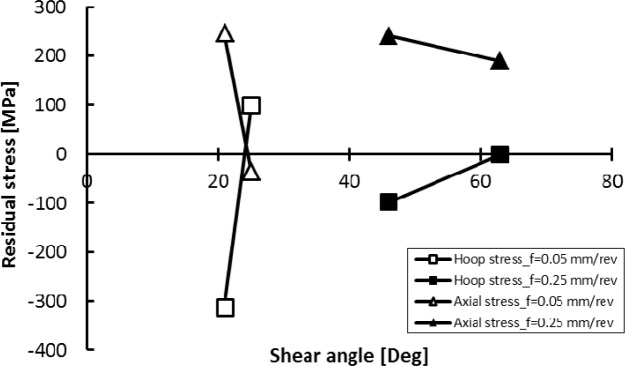
Effect of the shear angle on the surface residual stresses.

**Figure 21.**
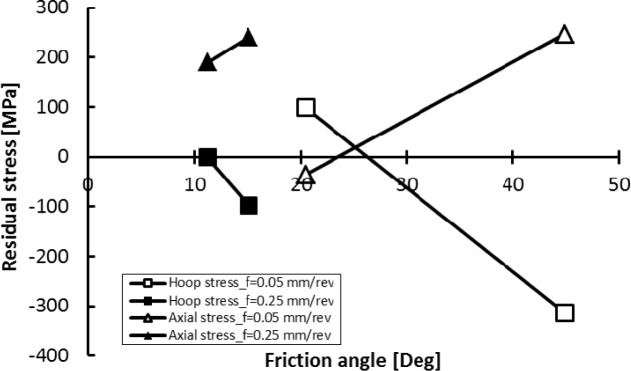
Effect of the friction angle on the surface residual stresses.

Figure 19 presents the effect of cutting speed on shear and friction angles. The cutting feed strongly affects the shear angle irrespectively of the cutting speed used. On the other hand, the shear angle is slightly affected when machining at low cutting feed, but it is significantly affected when machining at high cutting feed (Figure 19a). The reverse occurs for the friction angle (Figure 19b). Liu and Barash [32] stated that the larger the rake angle and/or the smaller the friction angle is, the larger the shear angle and consequently the better the surface quality. However, if one tries to describe the cutting phenomena with these geometric parameters, it is clear that as the shear angle increases (Figure 20); the surface stresses tend to be tensile in the hoop direction and compressive in the axial direction and this is true whatever the used cutting feed. This could be due to the triaxiality of the cutting conditions during orthogonal machining. When one looks at the friction angle as a describing parameter (Figure 21), it appears that the tendency is reversed. Interestingly, the trend (the slope) seems similar for both tested cutting feeds. As a final comment, it seems that to guarantee a good surface quality and compressive residual stresses, an optimization, in terms of the shear angle and friction angle, of the cutting conditions should be done.

## 3. Experimental Procedure

**Figure 22.**
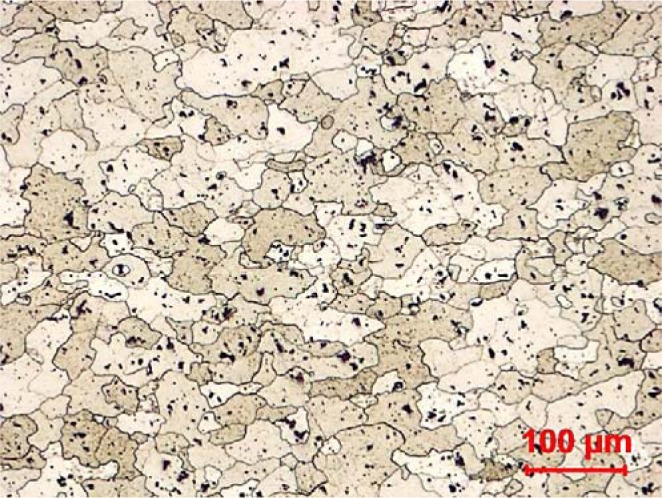
Optical microstructure of the aluminum AA7075-T651 alloy.

**Figure 23.**
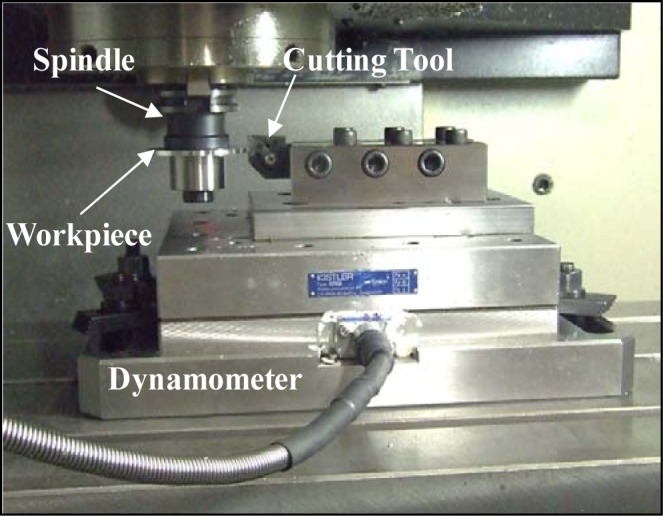
Orthogonal machining setup.

**Figure 24.**
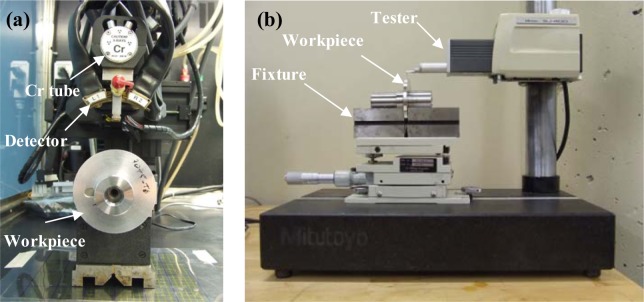
(**a**) Setup for residual stress; (**b**) surface roughness measurements.

Workpieces made of AA7075-T651 alloy were used. This precipitation hardenable aluminum alloy is widely used for the manufacture of aerospace and automotive structural components. The microstructure of the AA7075-T651 alloy is presented in Figure 22. The tested workpieces were disc-shaped, with a 70 mm external diameter, a 19 mm internal diameter, and a 4 mm thickness. Orthogonal tests were conducted on a NEXUS 410A 3-axis CNC machine (MAZAK, Florence, KY, USA) under dry cutting conditions (Figure 23). All cutting tests were performed with uncoated carbide inserts which referenced as TNMA120408 (K68 tool, Kennametal Inc., Latrobe, PA, USA). The inserts were mounted on a right hand tool holder, DTFNR2525M16KC04 (Kennametal Inc.), with a back rake angle of −5°. A newer cutting tool edge was used for each cutting condition in order to eliminate the effect of possible tool wear on the residual stresses. The specimens were machined at different cutting speeds and feeds, as shown in Table 3. The roughness parameters were measured using a Mitutoyo SJ-400 instrument (Mitutoyo Corp., Takatsuku, Japan) with a diamond stylus contact profilometer (Figure 24b). The cut-off was set to 0.8 mm and a Gaussian filter was used during the measurements.

**Figure 25.**
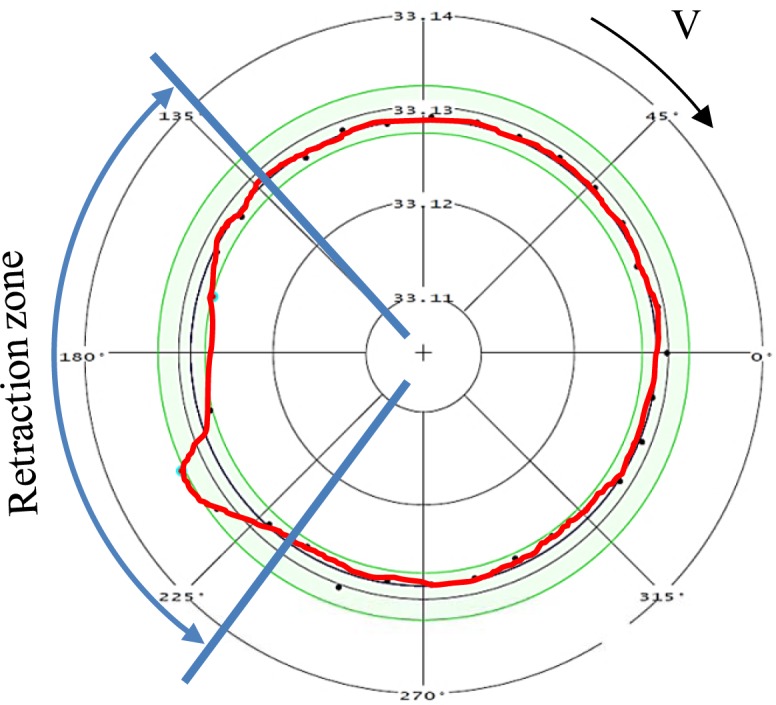
Circularity deviation of the machined workpiece for Trial #2.

During the retraction of the cutting tool, a part of the workpiece is machined with a cutting feed different from that programmed for a given cutting test. This change in the cutting feed is due to the deceleration of the cutting tool before the movement direction is changed during the retraction. Thus, in order to analyze the surface integrity of the machined workpiece, it was necessary to choose part of it to be representative of the cutting test. The representative zone was identified via the measurement of the circularity profile (Figure 25) using a coordinate measuring machine, BRIGHT STRATO 7106 (Mitutoyo, Aurora, IL, USA). The X-ray diffraction technique and classical sin2Ψ method were used for the residual stress measurements using a Proto iXRD^®^ system (Proto Manufacturing, Windsor, ON, USA) (Figure 24a), with a chromium tube. In-depth measurements were performed after the removal of the layer using electrochemical polishing. The residual stresses were measured in the circumferential direction (parallel to the cutting direction) and in the axial direction. Residual stress measurement provides the average stress in a diffracting volume defined by the size of the irradiated area and the depth of penetration of the X-ray beam (about 10 μm for 311 lattice plane in aluminum with Cr tube) [33]. The residual stress distributions produced by machining may vary significantly over depths of the same order of the X-ray’s penetration depth [34]. In the present work, the measured residual stresses were corrected using a commercial PROTO GRADIENT code. The cutting forces (tangential force, Ft, and thrust force, Ff) were measured using a Quartz 3-component dynamometer (Kistler, Amherst, NY, USA) (type 9255B) with the help of a Kistler charge amplifier (Kistler, USA).

## 4. Conclusions

This investigation showed the experimental results of the orthogonal dry machining of the AA7075-T651 alloy using an uncoated cemented carbide tool. The following are the significant findings of this study:

- The assessment of the surface finish shows that surface profiles displayed different features and are sensitive to cutting conditions in the axial as well as in the hoop direction.
- The formation of BUE was intensified by an increase in the cutting feed; however, an increase in the cutting speed reduced it and promoted the formation of the BUL on the rake face.
- The EDS analyses showed that the BUE and BUL have a dissimilar nature.
- SEM and EDS analyses showed that the primary origin of surface damage was the interaction between the tool edge and the iron-rich intermetallic phases present within the work material matrix.
- The hoop stress was predominantly compressive on the surface, and tended to be tensile as the cutting speed increased. The reverse occurred for the surface axial stress.
- The smaller the cutting feed, the greater the effect of the cutting speed on both axial and hoop stresses.

## Figures and Tables

**Table 1.** 2D roughness parameters.

Height (μm)	Amplitude distribution (μm)	
	Dimensional parameters	Dimensionless parameters
**Ra, Rq, Rpm, Rz, Rt**	Rk, Rpk, Rvk	Rsk, Rku

**Table 2.** Experimental results of the orthogonal machining.

Trial#	*F*_t_ (N)	*F*_c_ (N)	*l*_c_ (mm)	*t*_c_ (mm)	Φ (Deg)	β (Deg)
**1**	168	271	0.428	0.130	21	45
**2**	323	901	0.426	0.217	46	15
**3**	108	234	0.113	0.105	25	20
**4**	171	707	0.312	0.196	63	11

**Table 3.** Cutting conditions.

Trial #	Depth of cut, DOC **(**mm)	Cutting feed, *f* (mm/rev)	Cutting speed, *V* (m/min)
**1**	2	0.05	300
**2**		0.25	300
**3**		0.05	1000
**4**		0.25	1000
